# Photoconductive Dynamics of Photorefractive Poly((4-Diphenylamino)benzyl Acrylate)-Based Composites Sensitized by Perylene Bisimide

**DOI:** 10.3390/polym17010096

**Published:** 2025-01-01

**Authors:** Naoto Tsutsumi, Takafumi Sassa, Tam Van Nguyen, Ha Ngoc Giang, Sho Tsujimura, Boaz Jessie Jackin, Kenji Kinashi, Wataru Sakai

**Affiliations:** 1 Faculty of Materials Science and Engineering, Kyoto Institute of Technology, Sakyo, Kyoto 606-8585, Japan; kinashi@kit.ac.jp (K.K.); wsakai@kit.ac.jp (W.S.); 2 Photonics Control Technology Team, RIKEN Center for Advanced Photonics, Wako 351-0198, Japan; 3 Department of Materials and Life Science, Kyoto Institute of Technology, Graduate School of Science and Technology, Sakyo, Kyoto 606-8585, Japan; vantam32@gmail.com (T.V.N.); giangngocha@gmail.com (H.N.G.); sho.tsujimura@sekisui.com (S.T.); 4 Institute of Applied Science and Technology, Van Lang University, Ho Chi Minh City 700000, Vietnam; 5 Faculty of Chemical Technology, Ho Chi Minh City University of Industry and Trade, Ho Chi Minh City 72000, Vietnam; 6 Materials Innovation Laboratory, Kyoto Institute of Technology, Sakyo, Kyoto 606-8585, Japan

**Keywords:** photocurrent transient dynamics, photorefractive polymers, two-trapping site model, shallow trap, deep trap, hole mobility, initial transient photocurrent slope, quantum efficiency of photocarrier generation, photoelectron yield spectroscopy, density of states, ionization potential

## Abstract

The transient dynamics of photocurrents for poly((4-diphenylamino)benzyl acrylate) (PDAA)-based photorefractive (PR) polymers sensitized with perylene bisimide derivative N,N′-diisopropylphenyl-1,6,7,12-tetrachloroperylene-3,4,9,10-tetracarboxyl bisimide (PBI) at various composition ratios were studied. The PR polymer included (4-(diphenylamino)phenyl)methanol (TPAOH) photoconductive plasticizer and (4-(azepan-1-yl)-benzylidene) malononitrile nonlinear optical dye as well, which are needed for inducing PR effects. All the photocurrents measured at 640 nm were well simulated by a two-trapping site model considering photocarrier generation and recombination processes of the charge transfer (CT) complex between PBI and PDAA. The process of photocurrent simulation allowed for analyses of the dependences of hole mobility, quantum efficiency (QE) of photocarrier generation, trapping parameters, and recombination coefficient on the PDAA/TPAOH content. Finally, the PDAA content dependences of the trapping and recombination properties were compared with those of the PR parameters of the optical diffraction efficiency, optical gain, and response time.

## 1. Introduction

The photorefractive (PR) effect in a polymeric material with very tiny optical diffraction was first observed in 1991 [1]. Later, in 1994, optical diffraction of 86% with a high optical gain was reported in a PR polymer [2]. PR polymers consist of a photocarrier generator of holes and electrons along with an acceptor (sensitizer), a hole transport manifold (photoconductive polymer), a second-order nonlinear optical (NLO) dye that induces the Pockels effect, and a plasticizer to decrease the glass transition temperature of the PR polymer. In particular, an orientational enhancement of the NLO dye to increase the refractive index was introduced, which led to increases in the optical diffraction and optical gain [3]. Many studies on the PR trap density, space charge field, diffraction efficiency, optical gain, and time response have been reported for various types of PR polymers [4,5,6,7,8,9,10,11,12,13,14,15,16,17,18,19]. In recent studies, however, the fundamental photoconductive properties of photocarrier generation and hole transport, as well as the energetic aspects of molecules and composites, have not been well studied, although these properties and parameters significantly affect PR performance.

From the aspect of industrial applications, the development of three-dimensional (3D) displays is one of the attractive issues for the present information technologies. PR polymers are some of the good candidates of updatable holographic 3D displays [18]. Several approaches [20,21,22,23,24,25,26,27] have been proposed for updatable holographic displays. Peyghambarian’s group demonstrated updatable holographic 3D displays using a holographic stereogram technique with a PR polymer device (4 inch × 4 inch) in 2008 [20]. They showed 3D imaging had fast writing time and hours of image persistence [20]. Later, Peyghambarian’s group presented multi-color holographic 3D imaging using a high-frequency (50 Hz) pulsed laser system with a PR polymer device of 12 inch × 12 inch (30 cm × 30 cm) display size in 2010 [21]. They also demonstrated updatable full-color holographic 3D imaging with a PR polymer device in 2021 [18]. We also developed holographic 3D imaging using a PR polymer device in 2012 [22]. Real-time holographic imaging requires a PR response higher than the video rate (30 flames s^−1^, 33 ms flame^−1^). Video-rate holographic imaging was achieved with a tripheylamine-based PR polymer device in 2012 [23]. Sasaki et al. also demonstrated a real-time dynamic hologram with response time of 8 ms using a ferroelectric liquid crystal PR device in 2013 [25]. Recently, dynamic holographic imaging was demonstrated using perovskite nanocrystal-doped liquid crystal in 2021 [27]. In our recent studies, we have developed poly(triarylamine)-based PR polymer composites with hundred-microsecond responses [28,29,30]. Poly(triarylamine) PR polymer composites have high photoconductivity due to high drift mobility in the order of 10^−2^–10^−3^ cm^2^ V^−1^ s^−1^. High drift mobility is preferred to faster response time, but too-high photoconductivity leads to frequent dielectric breakdown in middle and high electric fields. Introducing a second trap species, we have succeeded to control photoconductivity in middle and high electric fields, and response times in the order of a few hundreds of microseconds can be achieved [28,29,30].

For another imaging application, nonlinear high-resolution imaging via an orientationally enhanced PR effect beyond the linear diffraction limit was reported [31]. This technique will provide subwavelength-resolution imaging applications in microscopy, etc. [31].

Recently, low-amplitude coherently coupled bright and dark soliton pairs were theoretically realized in biased guest–host photorefractive polymers [32].

PR performance was enhanced by controlling the ion-assisted space charge-limited current [33]. The internal electric field was increased by stacked charges injected from the electrodes. A high PR sensitivity of 16.7 cm^3^ kJ^−1^ and a device stability of up to *E* = 80 V μm^−1^ at 633 nm were achieved, which is comparable to that of the inorganic PR crystals Bi_12_SIO_20_ and Sn_2_P_2_S_6_ [33].

The analysis of transient photocurrents is a good way to investigate the PR properties of trapping and the recombination of hole carriers involved in PR processes. We investigated the transient photocurrents for poly((4-diphenylamino)benzyl acrylate) (PDAA)-based PR polymers sensitized by [6]-phenyl-C61-butyric acid methyl ester (PCBM), PDAA/(4-(diphenylamino)phenyl)methanol (TPAOH)/(4-(azepan-1-yl)-benzylidene) malononitrile (7-DCST)/PCBM [34,35]. The transient photocurrent was well analyzed by a two-trapping site model. In other words, the analysis of transient photocurrents provides detailed information on the quantum efficiency (*QE*) of photocarrier generation, the mobility of hole carriers, the recombination rates, and the trapping and detrapping rates for shallow and deep traps.

Recently, we designed new PDAA-based PR polymer composites sensitized by the perylene bisimide derivative N,N′-diisopropylphenyl-1,6,7,12-tetrachloroperylene-3,4,9,10-tetracarboxyl bisimide (PBI), PDAA/TPAOH/7-DCST/PBI, and evaluated the PR performance [36,37]. It showed quite different changes in the transient photocurrent over time through the introduction of PBI as sensitizers. The photocarrier was found to be generated from the charge transfer (CT) complex between PBI and PDAA or TPAOH [36,37].

In the present report, the transient dynamics of the photocurrent measured at 640 nm for PDAA PR composites sensitized with PBI are analyzed via the two-trapping site model [38,39]. The effect of the sensitizer PBI on the charge dynamics and PR effect is studied, and the results are compared with those of sensitizer PCBM [34,35].

## 2. Materials and Methods

### 2.1. Materials

PDAA, TPAOH, 7-DCST, and PBI were used as a host PR polymer, a plasticizer, an NLO dye, and a sensitizer, respectively. PDAA and 7-DCST was synthesized in our laboratory [26]. PBI was also synthesized in our laboratory [36,37]. TPAOH was synthesized in our laboratory [26,40]. The structural formulae of these compounds have been previously described [36,37]. The details of each compound are shown in previous papers [36,37]. We have already investigated the effect of the photoconductive plasticizer, TPAOH, and 2,4,6-trimethyl-*N*,*N*-diphenylamine (TAA) on photoconductive and PR properties [41]. Both have a triphenyl amine moiety in the compound, and TPAOH assisted the charge transport in PDAA, but TAA significantly suppressed the photocurrent to lead to inferior PR properties [41]. We selected TPAOH as the photoconductive plasticizer. 7-DCST is commonly used as an NLO dye.

### 2.2. Sample Preparation

In PR polymers, the glass transition temperature (*T*_g_) is important for PR properties. Thus, the considerable content of the plasticizer TPAOH should be included to control *T*_g_. Furthermore, in the present composites, the total amount of TPAOH and PDAA is fixed as 70 wt%. Thus, we determined the present ratio of TPAOH and PDAA. Usually, 20–30 wt% NLO dye should be included. NLO dye of less than 20 wt% depresses the PR properties. We determined 30 wt% of NLO dye. Too much sensitizer leads to an unfavorable increase in dark current, which gives rise to dielectric breakdown in higher electric fields. Thus, 0.1–1 wt% sensitizer is enough. In the present case, 0.1 or 0.6 wt% was selected. We prepared PDAA PR polymers doped with TPAOH, 7-DCST, and PBI at various composition ratios by weight. The 7-DCST content was fixed at 30 wt%. The PCBM content was 0.1 or 0.6 wt%. The content ratio of PDAA to TPAOH was varied from 45/24.4 to 30/39.4. The abbreviations PDAA45, PDAA40, PDAA35, PDAA30, and PDAA30/0.1 indicate PDAA/TPAOH/7-DCST/PBI with composition ratios of 45/24.4/30/0.6, 40/29.4/30/0.6, 35/34.4/30/0.6, 30/39.4/30/0.6, and 30/39.9/30/0.1, respectively. The details of the preparation method have been reported in previous papers [36,37].

### 2.3. Characterization

The PR properties of the composites were reported in previous papers [36,37]. The detailed methods for measuring the PR properties have been previously described [36,37].

The transient photocurrent was recorded at 40 Vμm^−1^ under illumination by a 640 nm laser with a light intensity of 140 mWcm^−2^. The details were given in previous reports [34,35].

Photoelectron yield spectroscopy (PYS) was performed with a Bunkokeiki BIP-KV202GTGK PYS instrument (Tokyo, Japan).

The absorption spectra of the films were recorded with a Shimadzu UV-2101PC absorption spectrophotometer (Kyoto, Japan).

### 2.4. Equations for Transient Photocurrent

Here, the transient photocurrent can be simulated and analyzed via the following nonlinear dynamic equations based on two trapping sites and a single photocarrier generation/recombination site [38,39]:

(1) $$J_{ph}=e\mu \rho E-eD\frac{\partial \rho }{\partial x}$$

(2) $$\frac{\partial \rho }{\partial t}=\frac{\partial N_{A}^{-}}{\partial t}-\frac{\partial T^{+}}{\partial t}-\frac{\partial M^{+}}{\partial t}-\frac{1}{e}\frac{\partial J_{ph}}{\partial x}$$

(3) $$\frac{\partial E}{\partial x}=\frac{e}{\varepsilon_{0}\varepsilon_{r}}\left(\rho +T^{+}+M^{+}-N_{A}^{-}\right)$$

(4) $$\frac{\partial T^{+}}{\partial t}=\gamma_{T}\left(T-T^{+}\right)\rho -\beta_{T}T^{+}$$

(5) $$\frac{\partial M^{+}}{\partial t}=\gamma_{M}\left(M-M^{+}\right)\rho -\beta_{M}M^{+}$$

(6) $$\frac{\partial N_{A}^{-}}{\partial t}=sI\left(N_{A}-N_{A}^{-}\right)-\gamma_{R}N_{A}^{-}\rho$$

where *J*_ph_, *e*, *μ*, *ρ*, *E*, and *D* are the current density, elementary charge, charge carrier mobility, charge carrier density, electric field, and diffusion coefficient, respectively. *ε*_0_ and *ε*_r_ are the permittivity in vacuum and the dielectric constant, respectively. *N*_A_, *T*, and *M* are the total number density of carrier generation site A, that of shallow traps, and that of deep traps, respectively. *N*_A_, *T*^+^, and *M* ^+^ are the number density of anions in site A, that of filled shallow traps, and that of filled deep traps, respectively. γ_T_ is the rate of trapping by the shallow traps; *γ*_M_ is that by the deep traps; *γ*_R_ is the recombination rate; *β*_T_ is the rate of detrapping from the shallow traps; and *β*_M_ is that from the deep traps. The photogeneration cross-section *s* is given by *s* = *α*λ*ϕ* ⁄(*hcN*_A_), where *α* is the absorption coefficient of the carrier generation site A. *ϕ* is *QE* for the carrier generation site A. *h*, *c*, *λ*, and *I* are the Planck constant, speed of light, wavelength of the illuminated light, and intensity of the illuminated light, respectively.

## 3. Results and Discussion

### 3.1. Transient Photocurrent

**Figure 1:**
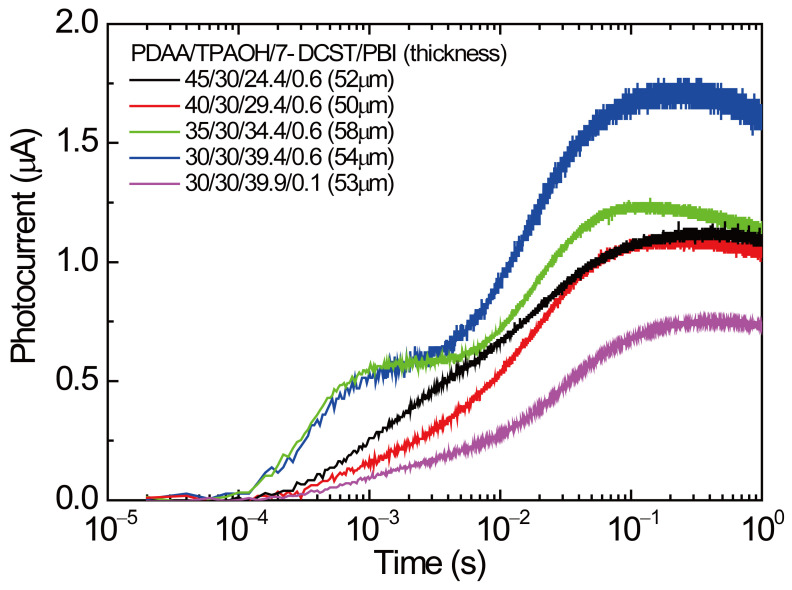
Transient photocurrents for PDAA45, PDAA40, PDAA35, PDAA30, and PDAA30/0.1. Key features, such as plateau regions or steep initial slopes, are confirmed for PDAA35 and PDAA30.

Figure 1 shows the transient photocurrent measured at 640 nm for the PR polymer composites with various ratios of PDAA to TPAOH and fixed ratios of 7-DCST (30 wt.%) and PBI (0.6 wt.%). In the figure, the photocurrent measured for PDAA30/0.1 is indicated as a reference. It should be noted that the photocurrent data were acquired using a picoammeter (6485, Keithley, Solon, Ohio, USA) with an electronic time constant of 120 μs. In the composites with 0.6 wt.% of PBI, PDAA30 and PDAA35 show a larger level in the time range measured against PDAA 40 and PDAA45. In addition, they show a plateau around a time of 10^−3^ s with a sharp initial slope, whereas PDAA 40 and PDAA45 show a monotonous increase with a mild slope. After the time of 10^−3^ s, all traces show the same trend: reaching a peak at around 10^−1^ s and then decaying gradually. The clear difference in the photocurrent level and the change around the time of 10^−3^ was obtained by changing the ratio of TPAOH to PDAA while the loading of the charge-generating sensitizer PBI was constant.

### 3.2. Hole Mobility and QE of Photocarrier Generation

For the simulation of the transient photocurrent later, the hole mobility *μ* and the product of photocarrier generation and absorption coefficient *αϕ* are needed to be individually evaluated [34,35]. The hole mobility can be obtained from PYS measurement and the quantum efficiency from the initial slope of the transient photocurrent.

**Figure 2:**
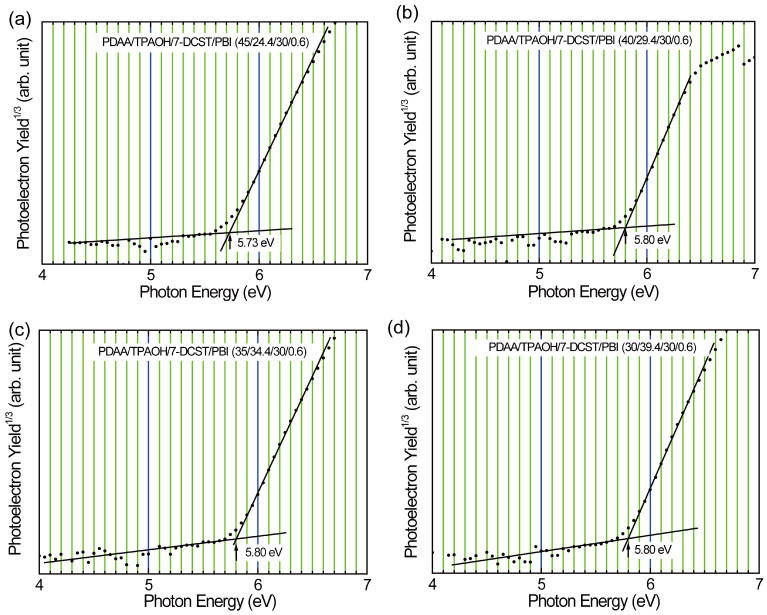
PYS spectra and ionization potential (*I*_p_) for (**a**) PDAA45, (**b**) PDAA40, (**c**) PDAA35, and (**d**) PDAA30. Dot is the PYS spectra. The intersection of slope and baseline is the ionization potential.

**Figure 3:**
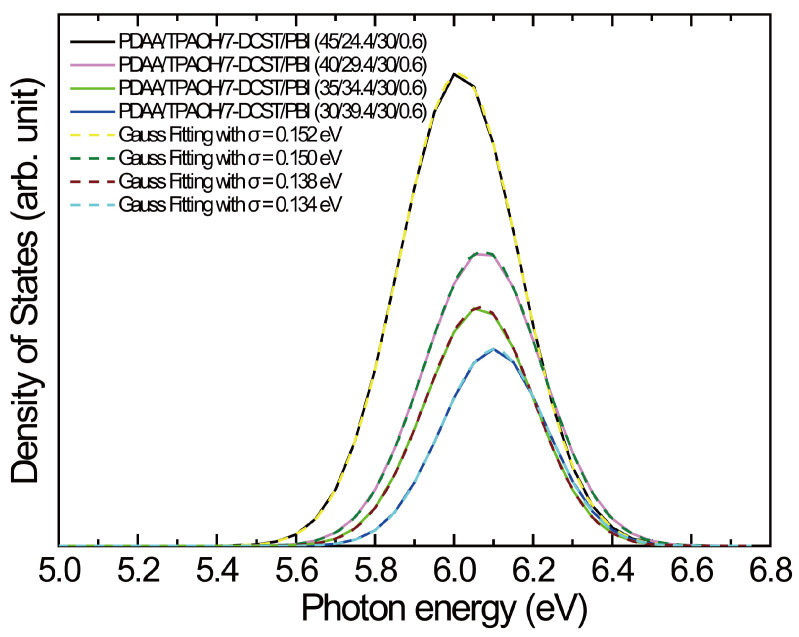
Gaussian peaks at the lowest photon energy extracted from the DOS spectra for PDAA45, PDAA40, PDAA35, and PDAA30. Dotted curve: Gaussian peaks fitted with the proper width of the DOS, *σ*.

PYS measurement provides the ionization potential (*I*_p_, highest occupied molecular orbital (HOMO) level equal to the negative value of *I*_p_) and the width of the DOS for the PR polymer composites. Figure 2 shows the plots of the photoelectron yield to the power of one-third vs. the photon energy. The intersection of the baseline with the initial slope in the photoelectron yield trace gives the ionization potential of the PR polymer composite. The ionization potentials are listed in Table 1. *I*_p_ values of 5.73 and 5.80 eV are measured, which are comparable with the values of 5.64 eV for TPAOH and 5.69 eV for PDAA [41]. A slightly higher *I*_p_ in the composite suggests the influence of 7-DCST (*I*_p_: 5.9 eV [41]). The first derivative of the photoelectron yield spectrum can give the DOS spectrum. Figure 3 shows the DOS spectrum of the separated Gaussian peak at the lowest photon energy for each composite processed with Peakfit software (Ver. 4). The lowest-energy DOS profile is characterized with a width of *σ*, indicating the degree of energetic disorder in the composites, and is summarized in Table 1. The DOS width decreases with increasing TPAOH content (decreasing PDAA content). This trend was found in PDAA/TPAOH/7-DCST/PCBM as well and attributed to the suppression of the dipole-trap effect caused by TPAOH with a smaller dipole moment of 1.774 D than that of 2.256 D for PDAA monomers [37]. It is interesting that in the current materials with PBI, a narrower *σ* is found in the lower content of PDAA by the replacement of PCBM, showing the positive effect of PBI on the reduction of the dipole-trap effect through the interaction with TPAOH.

The hole mobility in dispersive media is significantly affected by the energetic disorder. Considering the positional disorder as well as the energetic disorder, a universal law [42] for the mobility is obtained via Monte Carlo simulation as follows:

(7) $$\mu \left(E,T\right)=\mu_{0}exp\left[-{\left(\frac{2}{3}\frac{\sigma }{kT}\right)}^{2}\right]exp\left\{C\left[{\left(\frac{\sigma }{kT}\right)}^{2}-\Sigma^{2}\right]E^{1/2}\right\}$$

where *Σ* characterizes the positional disorder, *C* is an empirical constant, *μ*_0_ is the prefactor mobility, *k* is the Boltzmann constant, *T* is the absolute temperature, and *E* is defined above. Using the equation, hole mobility can be calculated with the evaluated *σ* and is listed in Table 1. In the calculation, *μ*_0_ = 0.01 cm^2^ V^−1^ s^−1^, *C* = 5.30 × 10^−4^ cm^1/2^ V^−1/2^, *Σ* = 3.6, and *E* = 40 Vμm^−1^ are used, following our previous study [34,35].

As discussed in the previous report [35], the slope of the initial transient photocurrent gives the *QE* of photocarrier generation because the trapping and recombination events are insignificant in the initial time range [39]. Thus, $\frac{dj_{photo}}{dt}{|}_{t=0}$ is expressed as follows:

(8) $$\frac{dj_{photo}}{dt}{|}_{t=0}=\mu eE\frac{\phi \alpha \lambda }{hc}I$$

where *μ*, *e*, *E*, *ϕ*, *α*, *λ*, *h*, *c*, and *I* are defined above. Considering the time constant of the used picoammeter (*τ*_RC_), the measured photocurrent *j*_τ_ (*t*) is related to $\frac{dj_{photo}}{dt}{|}_{t=0}$ as follows [35]:

(9) $$j_{\tau }(t)=\frac{dj_{photo}}{dt}{|}_{t=0}\left(t-\tau_{RC}\left[1-e^{-\frac{t}{\tau_{RC}}}\right]\right)$$

**Figure 4:**
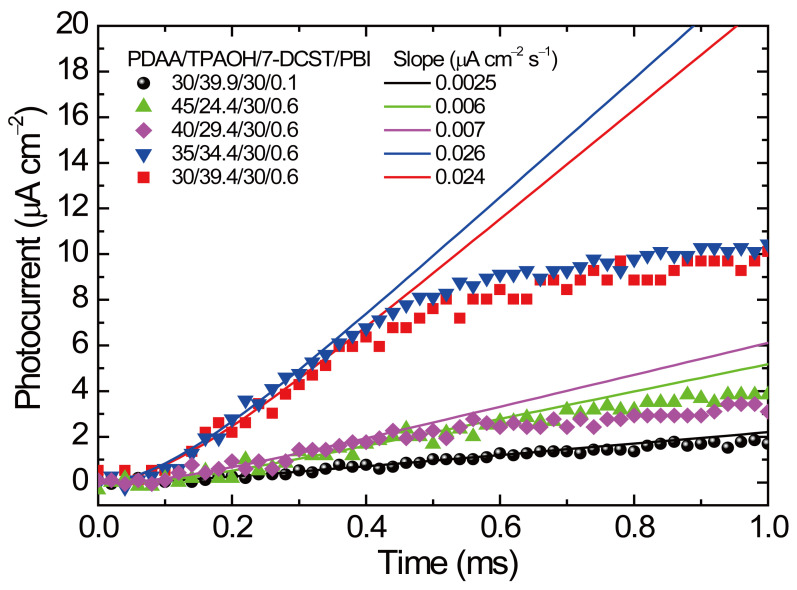
Transient photocurrent plots in the initial time region for PDAA45, PDAA40, PDAA35, PDAA30, and PDAA30/0.1. The solid lines are the curves fitted by Equation (9).

The initial transient photocurrents extracted from Figure 1 and the curves fitted by Equation (9) are shown in Figure 4. The values of $\frac{dj_{photo}}{dt}{|}_{t=0}$ measured from Equation (9) and of *ϕα* evaluated from Equation (8) are listed in Table 2. The values of $\frac{dj_{photo}}{dt}{|}_{t=0}$ for PDAA35 and PDAA30 are 3.4–4.0 times greater than those for PDAA45 and PDAA40. Notably, *ϕα* values of 0.723 and 0.762 are measured for PDAA45 and PDAA40, respectively, which are 5.6 and 6.0 times higher than the value of 0.128 for PDAA30/0.1. The ratio among these values coincides with the PBI content ratio of PDAA45 and PDAA40 to PDAA30/0.1, suggesting the relevance of monomeric PBI to charge generation sites in these samples. In contrast, *αϕ* values of 0.163 and 0.123 are measured for PDAA35 and PDAA30, respectively, which is much higher than the content ratio of PBI. To find out the origin of such high *αϕ*, the absorption spectra of PR composite films were investigated.

**Figure 5:**
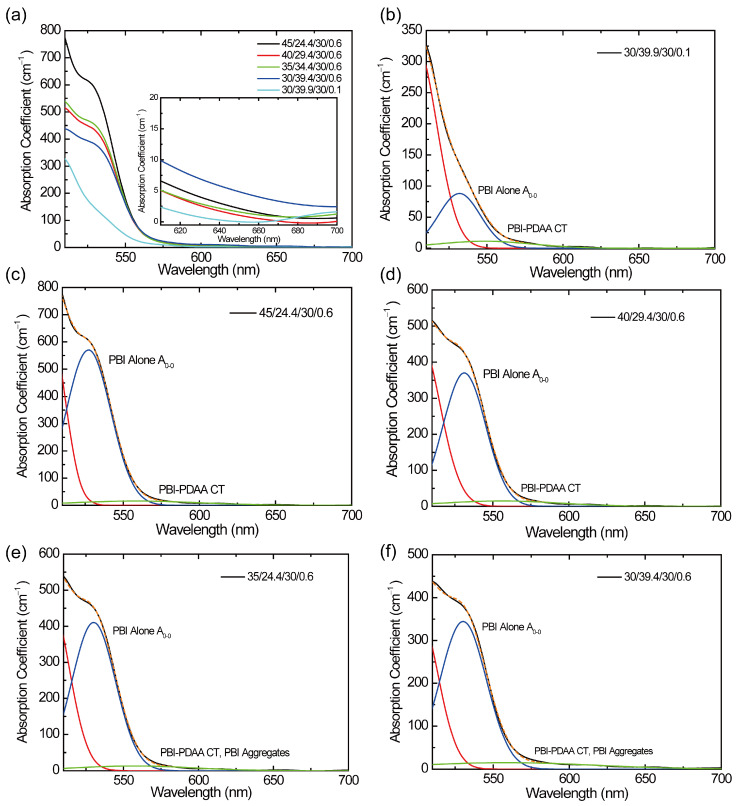
Absorption spectra and separated spectrum by Gaussian peak separation. (**a**) Absorption spectra of all composite films. The insert shows enlarged spectra in the wavelength range from 600 to 700 nm. (**b**–**f**) Separated Gaussian peaks. Blue spectrum: absorption due to 0–0 band, A_0–0_, of PBI alone. Red spectrum: absorption due to 0–1 band of PBI alone. Green spectrum: absorption due to PBI-PDAA CT complex and mixture of absorption due to PBI-PDAA CT complex and that due to PBI aggregates. Dashed orange spectrum: reproduced spectrum by summation of these spectra. (**b**) PDAA30/0.1, (**c**) PDAA45, (**d**) PDAA40, (**e**) PDAA35, and (**f**) PDAA30.

The absorption spectra of the composite films are shown in Figure 5a. Each spectrum can be separated into three Gaussian profiles with Origin software (Ver. 6.1). The results are shown for PDAA30/0.1 in Figure 5b, for PDAA45 in Figure 5c, for PDAA40 in Figure 5d, for PDAA35 in Figure 5e, and for PDAA30 in Figure 5f, in which the three profiles are represented by different color lines. We find that all spectra have similar Gaussian components characterized by the peak located around the wavelength of 500 nm (red), 530 nm (blue), and 550 nm (green). According to our previous study, the former two peaks are assigned, respectively, to the A_0-1_ band and A_0-0_ band of PBI, e.g., the peaks at 521 nm (A_0-0_), 488 nm (A_0-1_), 457 nm (A_0-2_), and 427 nm (A_0-3_) found in PBI films [37]. The last profile (green) extends from a wavelength of 420 to 680 nm and works as the photocarrier generation site for 640 nm excitation. We attribute the relevant molecules for this band to PDAA and PBI. PBI has a planar structure and easily forms an H-aggregate with face-to-face stacking geometry [43], which can be signified by the ratio of A_0-0_ to A_0-1_ in the absorption spectrum [40]. In a previous report [37], we reported a decrease in the ratio (A_0-0_/A_0-1_) in films compared with that in solutions, indicating the formation of PBI aggregates in films. Here, in this research, only a small amount of PBI is loaded in composites. It is reported, however, that aggregates of the perylene tetracarboxylic diimide (PDI) derivative, with a similar structure of PBI, are formed at a PDI content of 0.3 wt% and higher [44]. Moreover, in the sample preparation process here, the film is softened at a temperature of 140 °C under high pressure, which can accelerate the formation of aggregates. Such formed PBI aggregates are known to generate a new absorption band centered at a wavelength of 590 nm [44,45]. PBI aggregates allow for the migration of excitons and generate charges at the interface between donor sites [45], which suggests that a CT complex can be formed between the PBI aggregate and PDAA/TPAOH as well as between the PBI monomer and PDAA/TPAOH with extended optical absorption in the long-wavelength region. It should be noted that the role of PBI aggregates on exciton migration alters with the stacking arrangement of PBIs by changing the stability of excimers. [46,47] Thus, not all PBI aggregates formed in the composites can be involved in charge generation by photoexcitation. As a result, the broad absorption band centered at 550 nm in Figure 5 originates from PBI aggregates and the CT complexes between PBI monomer/PBI aggregates and PDAA/TPAOH. Particularly, only the CT complexes can work as photocarrier generation sites.

In Table 2, the absorption coefficient at 640 nm is listed. The value of *α*_640_ reaches a minimum at PDAA40 with the decrease in PDAA content except for PDAA30/0.1. It means that the content of PBI aggregates increases with decreasing PDAA content (increasing TPAOH content). The increase in TPAOH causes the enlargement of the free volume and assists the aggregation of PBI. Along with the result of *αϕ* above, we attribute the dominant site for charge generation in PDAA45 and PDAA40 as well as PDAA30/0.1 to the CT complex between the PBI monomer and PDAA/TPAOH, whereas in PDAA35 and PDAA30, we attribute it to that between the PBI aggregate and PDAA/TPAOH. The CT complex with PBI aggregates shows a large cross-section for photocarrier generation. A large *α*_640_ in PDAA45 can be partly from the non-charge-generating PBI aggregates.

### 3.3. Analysis of the Transient Photocurrent

Finally, estimating the number densities of the photocarrier generation site A, *N*_A_, and the trap densities, *T* and *M*, allows for the simulation of the transient photocurrent using the hole mobility *μ* listed in Table 1 and the product of the photocarrier generation and absorption coefficient *αϕ* listed in Table 2. For the trap densities, we used those reported for PDAA/TPAOH/7DCST/PCBM [34], since the dominant source for charge traps can be a conformational trap formed in the host polymer, PDAA. For *N*_A_, on the other hand, we used the following assumption. We have shown that the CT complexes between the PBI monomer/PBI aggregate and PDAA/TPAOH, four species in total, can be photocarrier generation sites in all composites. The number densities of those in each composite, however, are hard to estimate. Thus, we simply assumed that the total number densities for the composites with PBI of 0.6 wt% are just six times larger than those with PBI of 0.1 wt%, PDAA30/0.1. As shown above, the dominant photoexciting species in PDAA30/0.1 is the CT complex between the PBI monomer and PDAA/TPAOH. Here, using the molecular extinction coefficient (*ε*_M_, 7000 M^−1^ cm^−1^) reported for the intramolecular CT between perylene and triphenylamine moieties [48] and *α*_640_ measured (0.27 cm^−1^), *N*_A_ can be estimated as 1 × 10^16^ cm^−3^, resulting in 6 × 10^16^ cm^−3^ for PDAA45, PDAA40, PDAA35, and PDA.

An *α* value of 1.62 cm^−1^ (=0.27 cm^−1^ × 6) is assumed for PDAA45, PDAA40, PDAA35, and PDAA30, while the *ϕ* value can be evaluated and is listed in Table 2. Furthermore, as a reference, *ϕ*_PCBM_ for PDAA composites sensitized by PCBM [35] is also listed. *ϕ* values for the present PDAA composites sensitized by PBI are higher than *ϕ*_PCBM_ values for PDAA composites sensitized by PCBM. This means that the CT complex between PBI and PDAA is more preferred for charge carrier separation than that between PCBM and PDAA. In other words, a greater (or higher) stability of the CT complex has an advantage for charge carrier separation, which is significantly related to the smaller band-gap between the HOMO level of PDAA and the lowest unoccupied molecular orbital (LUMO) level of the acceptor [49]. The LUMO of PBI is assumed to be in the range of −4.0 and −4.2 eV from the absorption edge, whereas the LUMO level of PCBM is in the range of −3.7 [50], −3.8 [34,51], and −3.95 eV [52]. Furthermore, the utilization of the CT complex absorption band shows great merit in expanding the absorption region to the red and near-infrared regions for PR polymer composites. In addition, a very low absorption coefficient of the CT complex absorption band like the one in the present case promises bright holographic images due to the very low absorption of images by PR polymer composites.

**Figure 6:**
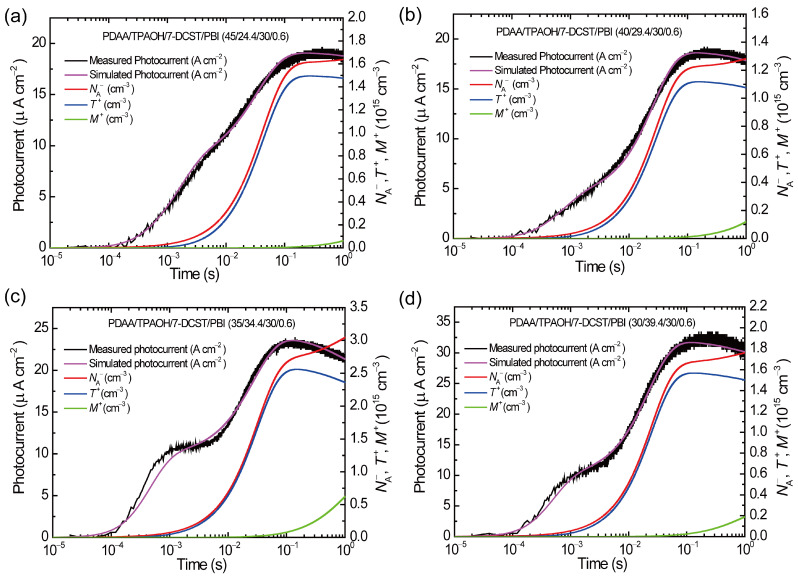
Comparison of measured and simulated photocurrents for (**a**) PDAA45, (**b**) PDAA40, (**c**) PDAA35, and (**d**) PDAA30. Black and pale curves: measured and simulated transient photocurrents, respectively; red curve: transient density of CT complex anions *N*_A_^−^; blue curve: that of the filled shallow traps *T*^+^; green curves: that of filled deep traps *M*^+^.

**Figure 7:**
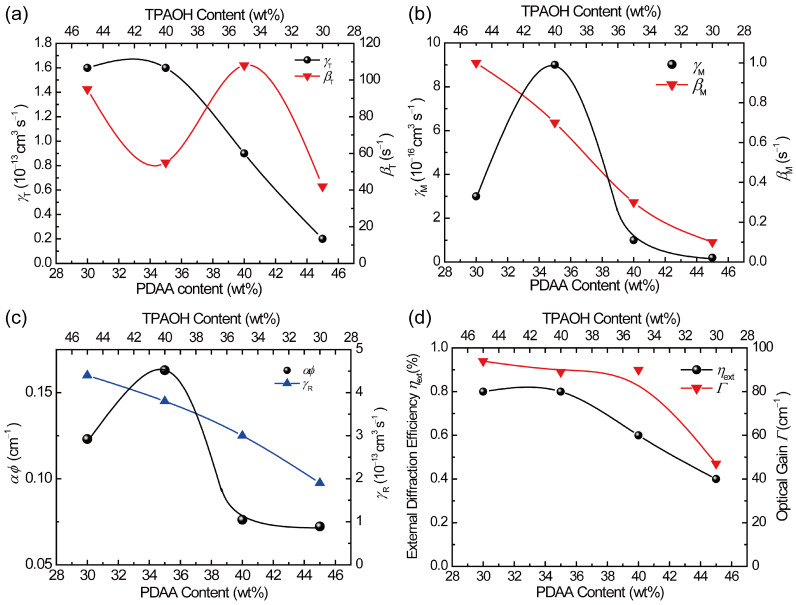
(**a**) PDAA (or TPAOH) content dependences of *γ*_T_ and *β*_T_, (**b**) those of *γ*_M_ and *β*_M_, (**c**) those of *αϕ* and *γ*_R_, and (**d**) those of the PR *η*_ext_ and *Γ* taken from our previous paper [37]. The solid lines are guides for the eye.

Simulated results of the transient photocurrent for composites with PBI of 0.6 wt.% are shown in Figure 6. All results reproduce the measured traces well, assuming a single photocarrier generation site, although some deviation occurred in the early time range in PDAA35. Sets of fitting parameters are listed in Table 3, where *T* and *M*, which are referred from the previous report [34], are included. To see the effect of the parameters on photocurrent traces with PDAA content, they are arranged with three properties in Figure 7: (a) shallow trapping (*γ*_T_, *β*_T_), (b) deep trapping (*γ*_M_, *β*_M_), and (c) recombination (*γ*_R_). Additionally, values of *αϕ* are also shown in Figure 7c. Here, we point out that the deviation in the early time range in PDAA35 can be compensated by the new two-trapping site model we proposed, in which two photocarrier generation sites are considered [35]. In addition to the CT complex, PBI aggregates would work as another photocarrier generation site for PDAA35.

The shallow trapping parameters represent the plateau in the photocurrent trace around 10^−3^ s. A larger *γ*_T_ and smaller *β*_T_ result in a clearer plateau region, yielding quicker filling of charge traps for PDAA35 and PDAA30. In contrast, PDAA45 and PDAA40 do not show a clear trapping effect. These results imply that the increased content of TPAOH (HOMO level: −5.64 eV) in PDAA35 and PDAA30 works as an effective shallow trap in the PDAA (HOMO level: −5.69 eV) hole transport manifold. Shallow trapping would reasonably play an important role in PR performance.

The transient photocurrent largely decreases in the time range after 10^−1^ s, which is ascribed to the significant increase in *M* ^+^. Namely, the shape of peaks around 10^−1^ s in the trace is determined by the parameters for deep trapping and recombination. Larger *γ*_M_ and larger *β*_M_ show sharper peaks with more abrupt decay due to deep trapping, which is furthermore assisted by a larger *γ*_R_ for PDAA35 and PDAA30. As was mentioned above, *αϕ* reflects the arrangement of PBI molecules. PDAA lower than 35 wt% is dominated by the complex with PBI aggregates giving a higher *αϕ*, whereas PDAA higher than 40 wt.% is dominated by the complex with PBI monomers giving a lower *αϕ*. A higher *αϕ* and larger trapping effect for PDAA35 and PDAA30 lead to larger modulation in the photorefractive refractive index, which is straightly related to higher external diffraction efficiency *η*_ext_ and optical gain *Γ* [37], as shown in Figure 7d.

Trapping and detrapping times are summarized for shallow and deep traps for all samples in Table 4. The time for residing in the both traps is longer for PDAA45 and shorter for PDAA35 and PDAA30. This means that the trap level for both traps is deep for the PDAA-rich sample, PDAA45, and shallow for the TPAOH-rich samples, PDAA35 and PDAA30.

The PR response time measured at 633 nm is compared with the trapping and detrapping times for shallow and deep traps in Table 4. The PR response time is slower than the trapping time for shallow traps and faster than that for deep traps. This result implies that shallow traps play an important role in the PR response time because the PR response time includes the time for space-charge formation after the charge carriers are trapped in shallow traps. The same tendency was reported for PDAA PR polymers sensitized by a PCBM sensitizer [35].

## 4. Conclusions

The transient dynamics of the photocurrents and PYS spectra for PDAA PR polymer composites sensitized by a PBI sensitizer were investigated. The *I*_p_ and DOS were evaluated via PYS for each PR composite. *I*_p_ values of 5.73 and 5.80 eV were measured. The DOS decreases with increasing TPAOH content. The hole mobility, estimated from the DOS, increases with increasing TPAOH content (decreasing PDAA content). The slope of the initial transient photocurrent gives the *QE* of photocarrier generation. The CT complex between the PBI monomer and PDAA/TPAOH is the source of photocarrier generation for all PR composites, and that between PBI aggregates and PDAA/TPAOH is added for PDAA35 and PDAA30. All transient photocurrents are simulated well with a two-trapping site model. A distinct plateau due to filling shallow traps appeared in the vicinity of 10^−3^ s for the TPAOH-rich samples, PDAA35 and PDAA30. Namely, TPAOH, possessing a slightly lower HOMO level than PDAA, works as an effective shallow trapping site in the PDAA hole transport manifold. The higher *αϕ* and larger trapping effect for PDAA35 and PDAA30 lead to larger modulation in the photorefractive refractive index, which is straightly related to enhanced PR performance with a higher external diffraction efficiency *η*_ext_ and optical gain *Γ.* The significant increase in the transient density of filled deep traps *M*^+^ contributes to the significant decrease in the transient photocurrent in the time range after 10^−1^ s.

## Figures and Tables

**Table 1:** Ionization potential *I*_p_, width of DOS, and hole mobility *μ*.

Sample	*I*_p_ (eV)	Width of DOS (eV)	*μ* (cm^2^ V^−1^ s^−1^)
PDAA45	5.73	0.152	2.87 × 10^−6^
PDAA40	5.80	0.150	3.18 × 10^−6^
PDAA35	5.80	0.138	5.52 × 10^−6^
PDAA30	5.80	0.134	6.76 × 10^−6^

*E* = 40 V μm^−1^, *C* = 5.3 × 10^−4^, *Σ* = 3.6, *μ*_0_ = 0.01 cm^2^ V^−1^ s^−1^.

**Table 2:** *dj*_photo_/*dt*, *αϕ*, *α*_640_, and *ϕ* for the samples corresponding to Figure 1. Values for *ϕ*_PCBM_ are taken from our previous paper [35].

Sample	*dj*_photo_/*dt* (A cm^−2^ s^−1^)	*αϕ* (cm^−1^)	*α*_640_ (cm^−1^)	*ϕ* (10^−2^)	*ϕ*_PCBM_ (10^−2^)
PDAA30/0.1	0.0025	0.0128	0.27	4.74	− *
PDAA45	0.006	0.0723	3.02	4.46	2.17
PDAA40	0.007	0.0762	1.73	4.70	4.23
PDAA35	0.026	0.163	2.25	10.1	8.05
PDAA30	0.024	0.123	5.82	7.58	4.09

* No data.

**Table 3:** Trapping and recombination parameters obtained via simulation fitting analysis.

Sample	*γ*_T_ [cm^3^ s^−1^]	*T, M* [cm^−3^]	*β*_T_ [s^−1^]	*γ*_M_ [cm^3^ s^−1^]	*β*_M_ [s^−1^]	*γ*_R_ [cm^3^ s^−1^]
PDAA30/0.1	0.45 × 10^−13^	1.43 × 10^16^	63	1 × 10^−16^	1	4.7 × 10^−13^
PDAA45	0.2 × 10^−13^	3.18 × 10^16^	42	2 × 10^−17^	0.1	1.9 × 10^−13^
PDAA40	0.9 × 10^−13^	1.58 × 10^16^	108	1 × 10^−16^	0.3	3.0 × 10^−13^
PDAA35	1.6 × 10^−13^	1.58 × 10^16^	55	9 × 10^−16^	0.7	3.8 × 10^−13^
PDAA30	1.6 × 10^−13^	1.43 × 10^16^	95	3 × 10^−16^	1	4.4 × 10^−13^

**Table 4:** Trapping and detrapping times for shallow traps and deep traps, with PR response times taken from our previous report [36].

Sample	(*γ*_T_ × *T*)^−1^ [ms]	*β*_T_^−1^ [ms]	(*γ*_M_ × *M*)^−1^ [ms]	*β*_M_^−1^ [ms]	Photorefractive Response Time [ms]
PDAA45	1.5	23	1560	10,000	555
PDAA40	0.7	9.3	625	3333	− *
PDAA35	0.4	18	70.3	1429	− *
PDAA30	0.4	11	233	1000	− *

* No data.

## Data Availability

The original contributions presented in the study are included in the article, further inquiries can be directed to the corresponding author.
